# ‘Something Was Missing’: A Qualitative Study of Parents' Expectations in Weight‐Related Health Care for Children

**DOI:** 10.1111/jan.70525

**Published:** 2026-02-09

**Authors:** Terhi Koivumäki, Marja Kaunonen, Susanna Anglé, Piia Jallinoja

**Affiliations:** ^1^ Health Sciences, Faculty of Social Sciences Tampere University Tampere Finland; ^2^ Hospital Services, Wellbeing Services, County of Pirkanmaa Tampere Finland; ^3^ Psychotherapy Service Mentor Turku Finland

**Keywords:** childhood obesity, health care, higher weight, parents, reflexive thematic analysis, weight stigma

## Abstract

**Aim:**

To explore how parents perceive health care encounters related to their child's higher weight and to interpret these experiences within the broader societal context shaped by cultural norms and representations.

**Design:**

A qualitative design was employed using semi‐structured interviews to capture parents' experiences. The study was informed by a conceptual framework that views weight as both a personal and socially constructed phenomenon.

**Methods:**

Eighteen parents from Finland were interviewed between May 2022 and June 2023. The data were analysed using reflexive thematic analysis to identify key themes in parents' experiences and perceptions.

**Results:**

Three main themes were identified. The relational dimension highlighted the importance of individualised care, where health care professionals take time to get to know the family and recognise each member as an individual. The emotional dimension emphasised the need to protect the child, strengthen parental self‐efficacy and provide sensitive, skilled support that fosters a sense of safety. The practical dimension focused on the need for active and targeted care, including structured weight‐related routines, multidisciplinary collaboration and services that respond to the everyday realities of families.

**Conclusion:**

The study highlights the need for health care encounters that are individualised, emotionally safe and sensitive to the diverse realities of families. Moving beyond weight‐centric approaches, care should offer a range of supportive options that reflect parents' varied expectations, concerns and needs.

**Impact:**

This study responds to the need for a deeper understanding of how parents experience health care encounters related to their children's higher weight. The findings highlight the importance of designing care interactions that provide professionals with adequate time, expertise and training to deliver individualised, stigma‐sensitive care.

**Reporting Method:**

COREQ.

**Patient or Public Contribution:**

No patient or public contribution.

## 1 Introduction

The global increase in paediatric higher weight (THL 2023; World Health Organization 2025) has resulted in more parents engaging with health care professionals (HCPs) to discuss and manage their child's weight. At the same time, there is a heightened emphasis shared by HCPs (Gutin 2022; Junno et al. 2019; Mäenpää and Vuori 2019) and parents (Saunders et al. 2023) on conducting these encounters in a more effective and sensitive manner.

Given the sensitivity surrounding children's weight, the language used in this article is intentionally respectful and non‐stigmatising. To avoid often medicalising and potentially stigmatising connotations associated with the term ‘obesity’ (Robbins et al. 2025), this article consistently uses the term ‘higher weight’. This terminology broadly refers to body sizes classified by the World Health Organization as overweight and obesity—defined as a body mass index (BMI) exceeding the age‐ and sex‐specific 85th percentile (World Health Organization 2025). The choice of language also reflects parents' preferences for more sensitive and non‐stigmatising terminology, with many preferring the use of ‘weight’ over terms perceived as clinical or stigmatising (Eli et al. 2022; Uy et al. 2019; Van Maarschalkerweerd et al. 2021).

Efforts to influence children's higher weight have primarily focused on modifying dietary choices and increasing physical activity (Ash et al. 2017). However, there is a growing recognition of the need for more comprehensive approaches that support positive parent–child relationships (Anderson and Keim 2016), strengthen parenting skills and assist families in managing socially and emotionally challenging situations (Ek et al. 2020).

A child's higher weight is not only a clinical question but also highly personal and dependent on body size‐related cultural narratives and discourses. These societal influences, including parental weight stigma, affect how parents perceive their child's weight and respond to health care recommendations. Therefore, a broader sociocultural lens is essential in paediatric weight‐related care (Bradbury et al. 2018).

To better respond to the increasing number of health care encounters around children's higher weight, it is essential to examine parents' expectations of these interactions and to consider them within their broader societal context.

## 2 Background

Parents play a pivotal role in managing the everyday circumstances associated with their child's weight (Harrison et al. 2025), including supporting adherence to treatment in health care (Steele et al. 2009). However, a range of factors may contribute to parents' hesitation in discussing their child's weight. Parents may not accurately perceive their child's weight status (Tompkins et al. 2015), which can lead to reactions of surprise, defensiveness, or even anger when HCPs raise concerns about the child's higher weight. Therefore, communication about weight should also consider parents' weight‐related feelings (Koivumäki et al. 2025). Parents often feel blamed or shamed for their child's weight in health care settings (Gorlick et al. 2021), and these emotions can sometimes lead to the avoidance of health care services (Puhl et al. 2011). Overall, parents express a desire for greater collaboration and communication from HCPs (Skantze et al. 2025). There is a clear need for further research to develop tools that support HCPs in building non‐judgemental, weight‐inclusive therapeutic relationships with families and children (Haqq et al. 2021).

Stigma by association, where parents are perceived as responsible for their children's weight, can significantly influence how they respond to discussions and their willingness to engage with recommended care (Davis et al. 2018; Gorlick et al. 2021). Parents of children with higher weight are often perceived as exhibiting lower‐quality parenting characteristics compared to those whose children are of a healthy weight, and they are frequently viewed as more responsible for their child's weight status (Patel et al. 2023). Moreover, parents themselves report feeling blamed and judged as inadequate caregivers (Davis et al. 2018).

While weight stigma and its health consequences have been increasingly acknowledged and studied among adults (Puhl et al. 2020), adolescents (Pont et al. 2017; Puhl and Lessard 2020) and children (Haqq et al. 2021; Pont et al. 2017), the specific phenomenon of parental weight stigma remains under‐researched and insufficiently recognised. Emerging evidence suggests that parents may experience weight stigma themselves, which can negatively impact their own well‐being, the well‐being of their child and the quality of the parent–child relationship (Lee et al. 2022).

HCPs, both generally (Puhl et al. 2016; Tomiyama et al. 2018) and within paediatric care specifically have been found to hold both implicit and explicit weight biases (Turner 2024). However, Turner (2024) also found evidence that HCPs are willing to address these biases, and various interventions and programmes have been developed to support them in discussing and managing paediatric weight issues more effectively with parents (Bonder et al. 2020; Castor et al. 2021). It is essential to consider the consequences of weight stigma holistically, taking into account both the children's and the parents' experiences (Haqq et al. 2021). Moreover, HCPs themselves report a lack of adequate tools and knowledge to provide effective and sensitive care for families dealing with a child's higher weight (Sjunnestrand et al. 2019).

In Finland, the public health care system and public health nurses therein play a key role in child weight‐related health care. Under the Health Care Act (1326/2010) ( Ministry of Social Affairs and Health 2010) and Government Decree 338/2011 (Ministry of Social Affairs and Health 2011), children under school age are entitled to at least 15 health check‐ups through public child health clinics, all attended by parents. Five of these are joint assessments, conducted by a nurse and a physician, focusing on the child's health and family well‐being (THL 2025a). In school health services, public health nurses meet annually with pupils. Comprehensive joint examinations are conducted in grades 1, 5 and 8 (ages 7, 12 and 15), with parents invited to participate. These assessments address both the child's health and the overall well‐being of the family (THL 2025b). Child health clinics and school health services in Finland are free of charge, with high participation rates. In 2023, health check‐up coverage reached 90% for children aged 1–2, 80% for those aged 3–6, and 72% for scheduled school health check‐ups conducted during the 2023–2024 academic year (THL 2024).

The discourse surrounding the child's higher weight remains largely stigmatising across various platforms, including traditional media (Quirke 2016), social media (Koivumäki and Jallinoja 2023), health care settings (Gorlick et al. 2021; Pont et al. 2017) and even official health care documents (Aamann and Erlik 2023). Thus, parents' experiences within health care settings reflect broader societal attitudes towards children's higher weight. This suggests that parental expectations should be understood within a wider sociocultural context, rather than being examined solely through the lens of health care.

## 3 The Study

### 3.1 Aim

This study aims to deepen the understanding of how parents experience health care encounters related to their child's higher weight, interpreting these experiences within broader societal discourses shaped by cultural norms and media. The findings seek to inform the development of more responsive and contextually aware health care practices.

### 3.2 Design

This study employed an interpretive qualitative design to explore how parents' past experiences shape their expectations of health care encounters related to their child's higher weight. While grounded in experience, the primary aim was to interpret these experiences within the context of broader societal discourses. Coding was not intended to be reliability‐checked, as this approach aligns with our use of reflexive thematic analysis (Braun and Clarke 2021), which supports a constructionist and critically engaged interpretation of participants' meaning‐making. These experiences are shaped by societal norms, values and personal histories, influencing how parents perceive, feel about, and respond to such encounters. Recognising that both weight and parenting are value‐laden in contemporary society, we adopted experiential and critical frameworks to guide our analysis. Drawing on a relativist and constructionist perspective, we approached parents' narratives as situated processes of meaning‐making. We recognised that broader societal discourses, particularly those surrounding a child's higher weight, shape these narratives, while still valuing the authenticity of parents' perspectives.

### 3.3 Ethical Considerations

The Ethics Committee of Tampere Region gave a supportive statement of the ethical acceptability of the proposed study (92/2021). The Consolidated Criteria for Reporting Qualitative Studies (COREQ) was followed (File S1) (Tong et al. 2007).

Regarding the ethical conduct of the interviews, the interviewer (first author) anticipated potential emotional responses and ensured openness by informing participants of the interview topic and their right to withdraw at any time. Her professional experience in working with parents and patients facilitated sensitive discussions. If distress occurred, she was prepared to refer participants to appropriate support services. Most parents valued the opportunity to reflect on their experiences with health care regarding their child's higher weight, though a few became emotional when recalling family circumstances during weight gain. The interviewer responded with empathy and active listening, and parents expressed appreciation for the safe, supportive environment.

In addition, the ethics review included a data protection statement detailing the processing and storage of personal data, which was shared with participants prior to the interviews. Paper‐based consent forms were stored in a locked cabinet accessible only to the researcher, while electronic data were kept in a secure, GDPR‐compliant cloud service requiring a username, password and two‐factor authentication.

### 3.4 Recruitment

Participants were recruited through purposive sampling. The recruitment drive included posts on the first author's social media accounts (Facebook, LinkedIn, Twitter), an interview in the regional newspaper *Aamulehti* featuring a call for participants, and an invitation published on the Finnish Heart Association's family website, *Neuvokas perhe* (the Smart Family). Interested parents contacted the first author directly by email and received study information, including data protection and consent procedures. Participants chose between face‐to‐face or Zoom interviews, with in‐person locations selected by the participant—ranging from homes to public spaces like library study rooms. Informed consent, either written or audio‐recorded, was obtained prior to each interview. Two pilot interviews were included in the final dataset, as they met inclusion criteria and required no changes to the interview guide.

Parents were eligible to participate if they had a child whose higher weight had been identified in a health care setting and the child had been living with the parent at the time the weight gain began. During the interviews, parents were asked to recall the age at which they first noticed an increase in their child's weight. In some cases, parents had more than one child with a higher weight (see Table 1).

**TABLE 1:** Data collection and participant characteristics.

	Interview duration (min)	Parent's age at the time of the interview	Child(ren)'s sex and age at the onset of weight gain	Interview location	Marital status	Education
1	25	Mother 49 years	Boy 10 years	Remote	Married	Secondary education^a^
2	37	Mother 44 years	Girl 10 years	Face‐to‐face	Married	Secondary education
3	31	Mother 49 years	Boy 10 years	Face‐to‐face	Divorced	Secondary education
4	38	Mother 49 years	Boy 8 years	Face‐to‐face	Married	University degree^b^
5	44	Mother 46 years	Boy 10 years	Remote	Married	University degree
6	58	Mother 55 years	Two boys, 8 years and 12 years	Face‐to‐face	Married	Secondary education
7	73	Mother 60 years	Girl 5 years	Face‐to‐face	Divorced	Secondary education
8	67	Father 63 years	Two boys, 10 years and 12 years	Face‐to‐face	Married	Secondary education
9	33	Mother 30 years	Girl 4 years	Remote	Married	Secondary education
10	32	Mother 32 years	Girl 7 years	Face‐to‐face	Married	Secondary education
11	40	Mother 38 years	Girl 13 years	Remote	Divorced	Vocational school^c^
12	60	Mother 49 years	Boy 10 years and girl 8 years	Remote	Married	Secondary education
13	64	Mother 46 years	Boy 10 years	Remote	Married	University degree
14	48	Mother 47 years	Girl 13 years	Remote	Divorced	Secondary education
15	100	Mother 31 years	Two girls, 10 years and 11 years	Remote	Married	Comprehensive school^d^
16	41	Mother 47 years	Two boys, 14 years and 6 years	Face‐to‐face	Married	University degree
17	70	Mother 37 years	Boy 11 years	Face‐to‐face	Divorced	University degree
18	51	Mother 51 years	Boy 6 years	Face‐to‐face	Married	Secondary education

^a^ Education following comprehensive school, including general upper secondary and vocational education.

^b^ Higher education qualification awarded by a university.

^c^ Post‐comprehensive school education focused on practical skills and trades.

^d^ Basic education typically covering ages 7–16 in Finland.

### 3.5 Researcher Characteristics

The female interviewer and the first author of this study hold a background as a public health nurse. During her master's studies, she conducted interviews with fathers, and in her role as a development manager in a national family health promotion program, she interviewed and collaborated with parents and HCPs. Her master's and PhD programs included training in qualitative research.

Most participants were previously unknown to the interviewer and had responded to the study invitation published in a newspaper article or shared by the interviewer on social media, for example in parent‐focused groups. However, three interviewees already knew the interviewer beforehand through mutual acquaintances. Their prior recognition of the researcher encouraged them to respond to the study invitation. Based on the interview announcement, all participants were aware of the research topic and the interviewer's professional background prior to the initial contact.

### 3.6 Data Collection and Data Analysis

The first author conducted individual semi‐structured interviews across different regions of Finland between May 2022 and June 2023, with the interview durations ranging from 25 to 100 min. An open‐ended and flexible interview guide was used to encourage rich narrative responses, especially those concerning family life and weight‐related health care experiences. Follow‐up questions were employed to clarify and deepen tentative themes. All the interviews were audio‐recorded, with brief notes taken during the sessions and transcribed verbatim. Supplementary field notes were added post‐interview to enrich the analysis. Each parent was interviewed once, but additionally, one participant submitted a written reflection after the interview, which was incorporated into the dataset. No children were present during the interviews. The transcripts were analysed using Atlas.ti 9 (www.atlasti.com).

This study employed reflexive, inductive thematic analysis, chosen for its flexibility and capacity to support interpretative engagement with qualitative data (Braun and Clarke 2021). This approach is particularly well‐suited to research in health and wellbeing, especially when the findings are intended to be accessible to audiences beyond academia, such as HCPs (Braun and Clarke 2014). In keeping with reflexive thematic analysis, data adequacy was judged by the richness of the dataset and its capacity to address the analytic aims, rather than through notions of saturation (Braun and Clarke 2021). The number of interviews was not predetermined; all individuals who volunteered were included. After approximately 15 interviews, no substantially new perspectives relevant to the study aims were identified, and three additional interviews were conducted to ensure that every willing participant could share their experiences and to confirm the adequacy of the dataset.

Reflexive thematic analysis enables both semantic coding, which captures surface‐level meanings expressed by participants, and latent coding, which facilitates the deeper interpretation of underlying patterns and assumptions (Braun and Clarke 2021). A reflexive stance was considered appropriate, acknowledging that researchers are not immune to weight‐related assumptions and biases, which may influence data collection, interpretation and analysis (Rowland and Conolly 2024). This approach supports a nuanced understanding of parents' experiences while also situating them within broader social and cultural contexts. In practice, the analysis followed a non‐linear, iterative process involving movement back and forth across the six phases of thematic analysis, as outlined by Braun and Clarke (2021).

After the interviews, the first author engaged in a thorough familiarisation process by repeatedly listening to the recordings and reading the transcripts with the aim of achieving immersion and critical engagement with the data (Braun and Clarke 2021). As familiarity with the data deepened, it became possible to pose increasingly analytical questions, such as why parents expressed themselves in certain ways. The initial coding identified descriptions of health care experiences and needs. The analysis then progressed towards interpretative depth, aiming to uncover underlying meanings (Braun and Clarke 2021, 2023).

Coding was conducted reflexively through ongoing dialogue and collaborative decision‐making. Instead of independent double coding, the authors discussed interpretations, refined codes, and resolved disagreements. Theme development was conceptual and interpretive rather than consensus‐driven and shaped by the authors' positionalities. This iterative process fostered a shared understanding and strengthened the credibility of the coding framework, in line with Braun and Clarke's (2021) reflexive thematic analysis. Coding was an extensive process due to the richness and complexity of parents' narratives. The interviews were often lengthy and included not only experiences related to weight but also broader aspects of family life and other health care interactions. Determining the final themes required considerable time, as the accounts contained numerous details and nuances. In some cases, a single sentence included multiple codes, each highlighting different important insights that contributed to the development of themes.

As an example of the coding process, the following quotation was analysed: ‘She somehow relieved us of that sense of guilt and normalised the situation by listening to what we shared about our child's two homes, our family life and the child's activities and hobbies’. From this excerpt, four codes were identified: the HCP listened to the family's situation; the HCP normalised concerns about the child's weight; the HCP reassured the parent; and the HCP alleviated the parent's sense of guilt. These codes were then organised into two themes: Individualised Encounter and Care (Relational Expectations), subtheme: See individuals in the family, and A Sense of Safety for the Child and Parenthood (Emotional Expectations), subtheme: Offer sensitive and skilled support.

Final coding decisions were made jointly to ensure coherence and analytical depth (Byrne 2022). The multidisciplinary author team brought a range of perspectives to both the research process and the analysis. The first author, AA, a doctoral student in nursing science and a former public health nurse, has developed a family‐focused lifestyle counselling method and trains professionals in delivering sensitive, weight‐related care. Co‐author CC is a professor of nursing science, while co‐author DD is a psychologist, psychotherapist and lifestyle counselling trainer. Co‐author NN, a professor of health sociology, contributed a sociological perspective to the study. The authors' diverse professional backgrounds complemented one another, with each contributing distinct expertise in weight‐related issues and parental engagement. Ongoing discussions throughout the analysis process were essential to developing a shared understanding of the data. All authors share a Finnish cultural background, which informed their contextual interpretation and helped situate the findings within the broader societal and health care landscape in Finland.

### 3.7 Rigour and Reflexivity

Rigour in this study was approached through the principles of reflexive thematic analysis (Braun and Clarke 2021), with a focus on methodological coherence, reflexivity and transparency. The analytic process was guided by a clear alignment between the theoretical framework, research aim and interpretative approach. Reflexivity played a central role, as researchers actively engaged with their own positionalities, assumptions and emotional responses to the data. Familiarisation involved repeated reading and listening to the interviews, fostering depth and nuance in interpretation. Analytic decisions were systematically documented to ensure transparency and traceability.

To meet the disciplinary standards of trustworthiness, credibility was supported through researcher triangulation and sustained engagement with the data. Transferability was enhanced by providing rich contextual information about the Finnish health care system, participant characteristics and relevant sociocultural factors. Dependability was addressed through a maintained audit trail of key decisions, while confirmability was ensured by grounding interpretations in the participants' narratives. Given the sensitivity of the topic, ethical considerations were prioritised. Particular attention was paid to respectful language and emotionally charged interviews were collaboratively discussed to support balanced and thoughtful interpretation.

Transcripts were not returned to participants for review, in line with the reflexive approach emphasising interpretation over validation (Braun and Clarke 2021). However, participants were informed that they could contact the researcher after the interview to add any forgotten details.

## 4 Findings

A total of 18 parents participated in the study: 17 mothers and 1 father, whose ages ranged from 30 to 60 years. Collectively, these parents had 23 children with a higher weight, including both girls and boys. The age at which the children's weight began to increase varied from 4 to 14 years. Parents' experiences with their children's weight issues spanned from the present to approximately 10 years prior. The participants' characteristics are summarised in Table 1. Parents reported experiences that primarily took place in child health care clinics and school health services, although some also reported visits to nutritionists. A few parents had interactions with specialised health care providers.

Three distinct themes were identified from parents' expectations regarding health care encounters: a relational theme highlighting the importance of HCPs recognising both individual family members and the family as a unique unit; an emotional theme, emphasising the need for emotional safety; and a practical theme, reflecting the desire for more integrated and coordinated care (Table 2). In the following, we present the three themes and their subthemes.

**TABLE 2:** The main and sub‐themes of parents' expectations for health care encounters in managing a child's higher weight.

Main themes	Individualised encounter and care (relational expectations)	A sense of safety for the child and parenthood (emotional expectations)	Care practices that meet the needs of the family (practical expectations)
Sub‐themes	Get to know our family See individuals in the family	Protect the child Strengthen parental self‐efficacy Offer sensitive and skilled support	Active and targeted care Weight routines and structures in health care Multidisciplinary work

### 4.1 Individualised Encounter and Care

This theme reflects a relational perspective, emphasising the importance of HCPs engaging with families as more than mere recipients of lifestyle counselling. Instead, families are understood as unique constellations of individuals whose needs and experiences are shaped by their personal backgrounds and life situations. Parents expressed a desire to seek solutions collaboratively with HCPs, provided that the process was grounded in the specific circumstances and needs of their own family. The relational theme includes two subthemes, presented in the following:

#### 4.1.1 Get to Know Our Family

The first subtheme highlights the parents' desire to be recognised as a unique family with their own history, values and resources. Parents emphasised that a personalised and respectful encounter required HCPs to listen attentively and seek to understand the family's everyday life. Being able to describe their family's daily life and circumstances in their own words was particularly important to parents, as it allowed them to present their situation on their own terms and counter potential assumptions.

Well, I think—perhaps also through the lens of my own work—that we should somehow try to look at the bigger picture. To consider what else is happening there [in the family]. That it's not only about what is eaten or not eaten. (Parent 17)

Parents emphasised the importance of HCPs understanding their family context and circumstances. Parents expected that the HCP would demonstrate both a willingness and a capacity to explore the family's overall circumstances.

It's kind of about understanding the broader context—like, what kind of situation the family is living in at that moment. That's probably something that should get more attention. (Parent 6)

Parents shared their experiences of discussions with HCPs concerning physical activity and nutrition. These conversations were often perceived by parents as too superficial and narrowly focused. Instead, they wished to be asked not only about eating and exercise, but also about the overall well‐being and resources of the family—a dialogue instead of one‐sided lifestyle advice.

How to start digging into the possible causes with the family. Maybe more could be done in that regard. It's not just about getting advice from the health clinic for 3‐year‐olds and 4‐year‐olds on what they should eat and how they should exercise. A more conversational approach would certainly be beneficial. (Parent 16)

Parents frequently regarded generic physical activity and dietary advice as irrelevant or poorly aligned with their family's specific needs. Some parents also questioned the relevance of information alone in improving the situation, because ‘it's not really about a lack of knowledge anymore’ (Parent 5). When receiving general lifestyle advice, parents often felt that their family's habits were already healthy enough and saw no need for change. Such advice also led to frustration, as parents felt they were not provided with any new or useful information that could support their family in addressing their child's weight‐related challenges.

Well, we did go to the dietitian, and I guess we got all sorts of materials from the child health clinic too. I don't recall receiving any specific guidance, just a bunch of brochures and papers, which I dutifully took home. Maybe I flipped through them at some point over coffee, but I didn't really take them in, because I already knew that our diet—when we wanted it to be—was healthy. (Parent 7)

#### 4.1.2 See Individuals in the Family

In the second subtheme, parents highlighted the importance of HCPs recognising the distinct roles and viewpoints of each family member. They noted that individual needs related to a child's weight may vary, depending on factors such as age, personality, previous experiences, or level of knowledge. One wish expressed by parents was that each child would receive personalised, child‐centred care and guidance, where the child is seen as more than his/her weight. Parents believed this was attainable when HCPs demonstrated a genuine interest in the child's unique characteristics, as illustrated in the following extracts.

The nutritionist was the first who started with something beyond weight. At first, we were asked, ‘Tell us what kind of child this is, tell us who you are and how you live’. (Parent 17)

It should be more about guidance and, of course, listening to the child to hear what she/he has to say, because when weight increases, there might be something else behind it. (Parent 12)

Recognition of children's uniqueness also means acknowledging that children within the same family can vary significantly, particularly in terms of growth patterns. Some parents described notable differences in weight‐related growth patterns among their children and recognised similar trajectories in their own or close relatives' growth histories. Many parents expressed a desire for a greater recognition of individual, hereditary growth patterns in the assessment of their child's development.

Not just look at the child now and think that he/she doesn't fit into the general mould. We should look more at the child's history and see that his/her growth has been like this all along, and it continues to follow that pattern. (Parent 15)

Understanding a child's history was seen by parents as essential for personalising care and ensuring that the advice provided was appropriate for the child's specific needs. Many parents also expected direct counselling for the child, tailored to age and individual circumstances. They emphasised the importance of assessing the child's motivation and some noted that children themselves should receive guidance on healthy lifestyle habits.

I don't think this should be addressed only to adults. Maybe more so that the child and the nutritionist could be involved, and the matter could be discussed at the child's level. (Parent 12)

Parents hoped that they too would be approached in ways that acknowledged their unique circumstances and emotional experiences. They stressed the importance of recognising their existing knowledge and situation as the foundation for providing relevant guidance. This was exemplified by one parent, who sought professional support not only to address feelings of helplessness, but also to foster healthier everyday habits within the family.

I'm quite lost, I probably need comprehensive support on how to start addressing the issue. If someone could give specific instructions on how to eat or exercise or something. (Parent 14)

Some parents emphasised the importance of engaging both parents, particularly in situations where they were living apart. A few mothers noted that they were already familiar with the information provided by the HCP and suggested it would be more beneficial for the father to receive this information directly from the HCP, rather than through them.

So I told the school nurse that it would actually be good if my spouse was the one to be called this time, because we've already discussed these matters, was it a year ago? (Parent 13)

### 4.2 A Sense of Safety for the Child and Parenthood

This theme captures the deeply emotional undercurrents of parental expectations in care for children with higher weight. It reflects parents' longing for a care environment where both they and their child feel seen, heard and supported without judgement. This theme is inherently emotional, encompassing parents' desires to protect their child from stigma, to feel competent and empowered in their parenting and to receive sensitive, skilled support that acknowledges the emotional complexity of navigating their child's weight‐related challenges.

#### 4.2.1 Protect the Child

The most cited aspect among the parents was ensuring the child's sense of security during the health care visits, particularly in relation to self‐confidence and body satisfaction. Parents hoped that their child would not be blamed for his/her body size, behaviour, or choices. They strongly believed that any form of intimidation could be harmful to the child, even though they acknowledged that identifying the safest approach is not always straightforward.

[it is] such a delicate balancing so that no one feels blamed or accused, or gets the impression that there is something wrong with them. (Parent 6)

Blaming is the worst part. And pointing out that if you eat sugar or such, it shouldn't be done in any way. (Parent 12)

Parents believed that the responsibility for managing their child's weight and related health care procedures should rest with adults, including both themselves and HCPs. They also felt it was their duty to safeguard the situation on behalf of their child. Ensuring that HCPs were perceived as safe and trustworthy was crucial to parents, and this was sometimes achieved by meeting the professionals in advance to establish trust before involving the child. This fear reflects parents' concerns that HCPs might, intentionally or not, cause emotional harm to the child by implying blame or deficiency. It also indicates uncertainty about professionals' abilities to approach the topic with sufficient sensitivity.

Well, I somehow feel that when I kind of vouch for that person, that they won't blame the child—it really highlights how, if the child has managed to live up to this point fairly carefree, without worrying too much about the issue [the weight], without feeling guilty or hating themselves, then if some unfamiliar adult comes along and somehow signals that there's something wrong with them… That's maybe where the sense of safety lies—in knowing what kind of person will be talking about the issue. (Parent 17)

Parents suggested that weight‐related discussions should take place without the child being present. This would allow adults to speak openly without worrying about the potential impact on the child's well‐being.

And the fact that this weight issue is preferably discussed among adults, rather than the child going alone to the nurse and the nurse telling the child that they have a bit too much weight for their height. (Parent 15)

Parents did not want their child to be treated differently because of his/her higher weight. They expressed relief when, for example, other children were given similar tasks, such as completing a food diary, as one parent stated, ‘it was quite comforting…someone else was doing it [the food diary] at the same time’ (Parent 18). This wish reflects the parent's desire for their child to be perceived and treated like any other child, or at least not singled out. It highlights how differentiation based on weight is experienced as stigmatising. At the same time, it reveals a protective instinct, as parents seek to shield their child from potential harm, such as bullying or exclusion, that may arise due to weight‐related differences.

Overall, parents highly valued and desired positive feedback and encouragement—not only for their child's choices but also for their character. Like one parent hoped to hear: ‘what a nice, open, talkative child’ (Parent 6). It was particularly important that HCPs provide affirming and supportive feedback directly to the child. ‘That we focus more on what you're doing right, especially with children’ (Parent 11). Once again, parents expressed a desire for their child to be seen as a person in his/her own right, rather than being evaluated through the lens of weight.

Parents described challenges in discussing weight‐related issues with their child, particularly when explaining the need for additional health care visits. Sensitive language was seen as crucial for protecting the child's self‐esteem and parents expected the same sensitivity from HCPs.

And then the visit to the nutritionist is concerning in the sense that we haven't mentioned it [the visit] to our son at any point. We discussed this with the nurse, and she is also very sensitive about it and supports not talking about having excess weight or anything like that. (Parent 13)

Some parents, particularly those with personal or familiar vulnerabilities, also voiced concerns that addressing weight could inadvertently trigger disordered eating. Parents struggled to weigh the potential harms associated with higher weight against the risk of triggering an eating disorder. They found it difficult to determine which posed a greater threat to their child's well‐being.

I'm a bit lost with this because my sister has had bulimia. And maybe it's something that has come from home, and I also notice that sometimes I mention weight, even though I know I shouldn't. So, I'm a bit lost in that sense, as I've always tried to get my daughter to join me in physical activities, like going for a walk or something. (Parent 14)

#### 4.2.2 Strengthen Parental Self‐Efficacy

Parents described a range of emotions, such as fear, hopelessness and inadequacy, that emerged during health care discussions about their child's weight—often undermining their sense of parental competence. Although they acknowledged that HCPs often attempted to approach the topic with sensitivity, these encounters still frequently elicited negative emotional responses. As one parent shared: ‘It was distressing for me in the sense that, already at the health clinic, they always kept asking how we eat at home’ (Parent 7). Some parents recognised that HCPs had the capacity to enhance parental self‐efficacy and provide a sense of relief. Parents particularly appreciated opportunities to openly discuss difficult emotions, such as shame, with HCPs.

And for me it was wonderful when I visited the nutritionist, and then she talked to me about my shame. Even though we were there to talk about my son's issues, we ended up discussing my situation as well. (Parent 17)

Feelings of guilt and perceived blame were commonly expressed in parents' accounts. While some parents felt that HCPs attributed responsibility for their child's weight to them, others recognised that their guilt stemmed from internalised beliefs and self‐judgement. These experiences illustrate how parents may come to view their child's weight as a reflection of their own success or failure in parenting.

Maybe the phone call was actually quite appropriate, and the public health nurse was professional. It was more about my own reactions. Maybe it struck a nerve related to feelings of inadequacy or not knowing what to do—like, okay, we already talked about this last year, and now the situation is just as bad. (Parent 17)

Being seen and heard as actively involved, competent parents was important to participants. They wanted to demonstrate their efforts and healthy choices, often highlighting the strategies they had tried, the knowledge they held and the values guiding their actions. Parents felt compelled to adopt a defensive and explanatory stance when their child's weight was discussed. One parent, for example, described trying to reassure the HCP about her nutritional choices for the family.

I remember how I passionately talked about how we use oil and don't use butter and haven't seen cream except at Christmas and things like that. (Parent 6)

Parents' accounts revealed a sense of strong and proactive parenting, particularly in situations where they felt their child was entitled to appropriate care. Many described taking the initiative to secure support and medical evaluations from the health care system. Some parents felt that they even needed to be assertive, or at times, demanding, in order to get care for their child's weight‐related concerns. As one parent put it: ‘You have to be strict for the child's sake’ (Parent 11).

#### 4.2.3 Offer Sensitive and Skilled Support

Parents had a range of expectations regarding HCPs' behaviour and competence, but above all, they valued sensitivity in addressing their child's weight. They appreciated empathic interactions, characterised by attentive listening, gentleness, encouragement and reassurance. The following parent described positively one HCP that had met these expectations:

Well, she somehow took away our guilt and normalised the situation by listening to our child's experiences with two homes, our family life, and the child's activities and hobbies. She said that there was nothing to worry about. (Parent 17)

Some parents called for a shift in health care attitudes towards weight‐related issues, while others noted positive changes already underway—especially in counselling around eating and physical activity. These views were shaped by their own childhood experiences or past interactions with health care, including those involving older children within the family.

I was somewhat overweight as a child, and back then, the school nurse used to point it out in a different way than they do nowadays. (Parent 9)

The language and wording used by HCPs influenced how parents experienced the situation. Parents expressed specific preferences regarding how weight‐related issues should be discussed and what terminology should be used, particularly when speaking with the child. Positive and compassionate language was appreciated, whereas overly medicalised or blunt expressions often made the situation feel uncomfortable for parents.

I would give advice that weight should be discussed, not as BMI or other strange indicators or letter combinations, like saying the body mass index is now so high or something. (Parent 15)

In addition to empathetic behaviour and language, parents emphasised the importance of HCPs' competence in fostering a safe environment for discussing their child's weight. Competence was reflected in both professional experience and the approaches used to address weight‐related concerns. Some parents also emphasised the value of continuity, having regular contact with the same professional, which helped build trust and enabled open conversations.

The school nurse is the one who is closest, as doctor's check‐ups are quite infrequent. And if you think about it, there is a long relationship, for example, in elementary school where you see them for six years. So, it [the guidance] should come from there. (Parent 2)

Only one parent explicitly mentioned an HCP's personal values regarding weight. This professional was perceived as genuinely believing that having higher weight does not inherently reduce quality of life—a view reflected in her focus on the child's well‐being, joy and social relationships rather than weight alone. The parent experienced this approach as respectful and affirming, suggesting that the professional's values positively shaped the encounter.

I think the nutritionist's difference in attitude was because she genuinely believed that it's not the end of the world if a mother is obese or if a person is overweight in general. You can live a good life; it sounds like your family is living a good life, your child is having fun and is loved and has friends. So, it doesn't matter if there's overweight. (Parent 17)

### 4.3 Care Practices That Meet the Needs of the Family

The third theme reflects parents' expectations for concrete, structured and responsive care that fits their everyday lives.

#### 4.3.1 Active and Targeted Care

Many parents reported that the care they received felt overtly passive, or delayed, with HCPs responding too slowly to their concerns. These parents expressed a desire for more proactive and timely interventions from the outset. In some cases, parents appeared more concerned about their child's weight than HCPs, revealing a divergence in perspectives and priorities regarding the issue.

But I had already expressed my concern about the girl's weight gain back then. But at that point, the nurse had looked and said there was no reason to worry about it or any need for check‐ups or anything. (Parent 14)

In addition, parents described weight‐related care as superficial. They noted that HCPs often limited their involvement to monitoring the child's weight without offering further guidance or support, which left parents feeling confused. Many assumed that if the child's weight is being tracked, it should lead to concrete action within health care, as the following parent shared.

And I think maybe the strangest thing is that it's implied that the weight is high, but then nothing happens, even after seeing the nutritionist. And when no reason [for the higher weight] is found, it's just left at that. (Parent 18)

Parents frequently described the advice or support they received as either ineffective or overly generic. Many felt that the information provided was already familiar and did not significantly enhance their understanding or ability to address their child's weight. In some cases, communication from HCPs was limited solely to notes or growth curve charts, thus lacking further explanation or actionable guidance. For many parents, growth monitoring was viewed as a vital component of their child's overall health, not merely a routine procedure conducted during scheduled check‐ups.

They [HCPs] might have sent those papers indicating that the health check‐up was done, and then the weight percentages showing how much it has increased. But my recollection is that there wasn't any actual follow‐up contact from them. (Parent 2)

Parents most often wanted concrete and practical guidance to support everyday changes. This also reflects the parents' willingness to take action, provided that the guidance is compatible with their everyday life.

Concrete tips would definitely help better, like what I could offer as a snack after daycare or what would be a good power food that provides energy but not too much fat. It's something that others think too, and surely many other mothers ponder almost every day about what to make for dinner. (Parent 9)

As mentioned earlier, most parents valued sensitive communication to ensure a safe and supportive interaction. However, a few parents preferred more direct, fact‐based discussions about the health impacts of weight—even if difficult to hear—emphasising that it was the HCPs' responsibility to provide such guidance. This highlights the emotional complexity parents faced in balancing the risk of emotional harm with the need for health‐related understanding. This tension suggests that, while some parents consider information about the health consequences of higher weight essential for addressing their concerns, others believe such information is already well understood or worry that sharing it—particularly in the child's presence—could be harmful.

But on the other hand, I think it would be responsible to mention that being significantly overweight can have consequences, as the body might not function healthily. And those consequences can appear in the long term, which is why they are difficult. (Parent 6)

Experiences of passive care from HCPs left parents feeling isolated and unsupported in managing their child's weight. A common sentiment among parents was that, although the care they received was generally adequate, it did not effectively address or support the family in managing the weight issue.

And then we got a new appointment with the nutritionist six months later, and we discussed the same things again. The answers were the same, and we were really left alone with the issue. The only advice from health care was to walk a bit more or something like that. We didn't really get any support for it. (Parent 11).

We received a lot of good service and interactions. They tried, but maybe in the wrong way, as something was missing. (Parent 7)

#### 4.3.2 Weight Routines and Structures in Health Care

Parents described neutrally a range of health care routines, including the use of growth charts, regular appointments with public health nurses, weight control consultations, laboratory tests and referrals to nutritionists. However, parents also expressed ambivalence towards these routines. While they recognised them as part of health care's long‐standing and largely unquestioned traditions, they also voiced critical views of the weight‐centred approach embedded in these practices.

In Finland, we're quite chart‐oriented and often focus on whether someone fits into the expected mould. There's the middle curve [of weight], and we follow that, and if not, then it's something else. (Parent 16)

One routine aspect of care described by parents was the guidance they received on lifestyle habits. They recalled various HCPs offering advice on healthy eating and physical activity, often accompanied by brochures or other informational materials to take home. According to many parents, it is the role of health care to provide information about healthy lifestyles and this approach was seen as a natural part of routine weight monitoring.

But we just talked about how, yeah, more vegetables and other things, and fewer treats and so on. (Parent 13)

But indeed, it was at the age of 5, and then there was the visit to the nutritionist, where we had the diaries and filled them diligently. Then they said to review it again after some time, and at that point, it was already suggested to add more vegetables. (Parent 18)

Some parents observed that existing health care structures did not optimally support effective weight management or facilitate parental involvement. Challenges included the limited availability of health care personnel, inconvenient appointment times, and age‐related restrictions that limited parental participation.

#### 4.3.3 Multidisciplinary Work

Parents identified several unmet needs that they believed could have been addressed with support from a broader range of professionals. These needs often related to assistance that would have supported the family's daily life or strengthened their mental resilience.

So, it would definitely be good that if the nurse doesn't have enough time or expertise, she would refer [the client] to the next specialist. Because we are building the basic building blocks of the child's life when they are that age. (Parent 10)

Parents expressed a need for emotional support from professionals, recognising that psychological challenges such as mental fatigue and life stressors could affect both their child's weight and the family's overall ability to cope. However, the level of psychological and emotional support they required often exceeded what health care services were able or equipped to provide.

We might have needed that [psychological support] more than the visits to the nutritionist. We might have needed something like emotional guidance. (Parent 7)

Another form of support that parents hoped for involved assistance with everyday family life, including family dynamics and relationships. Parents acknowledged that this type of support is not always easy to provide, nor did they necessarily expect that the HCP they were currently seeing would be the one to address such issues.

The nurse at the child welfare clinic might not necessarily be able to sort out what's going on, what it's like at home, what's happening there. It would rather require something [similar] like, at least from my perspective, what we received when we've been to the family counselling centre about these things. (Parent 16)

While many parents reported being routinely referred to nutritionists, some expressed disappointment at not receiving such referrals despite wanting them. Nutrition consultations were generally perceived as a standard component of weight‐related care, something parents were entitled to, even when they did not have any specific needs regarding their child's dietary habits or nutrition. Additionally, although parents reported receiving advice on healthy eating and physical activity from HCPs, they felt that more practical and diverse support was needed. This included practical information on portion sizes and suitable snacks for the child, as well as encouragement and resources to support their child's participation in physical activities.

Probably the most help would have been if there was some kind of activity in the area that one could easily join to do sports or exercise, something that isn't like a competitive ice hockey team but more casual and suitable for people of all shapes and sizes, and not very demanding in terms of commitment. That would probably be quite necessary. (Parent 5)

In part, parents' expectations reflected a desire to externalise the weight issue beyond the responsibilities of parenting. They sought an external resource that could succeed in areas where they themselves had not found solutions, for example, facilitating their child's participation in physical activities. A few parents also mentioned the need for peer support, particularly for their children.

## 5 Discussion

The aim of this study was to examine how parents, drawing on their past experiences, expect health care to engage with them in the context of their child's higher weight. These expectations were shaped not only by individual encounters with HCPs but also by prevailing cultural narratives surrounding the child's higher weight, parenting and health. Three types of expectations were identified: relational, where parents wished to be acknowledged and respected as unique individuals; emotional, where they sought a sense of safety and support; and practical, where they expected effective and coordinated care. In the following, these main findings will be discussed.

First, the parents involved in this study emphasised the need for individualised support that reflects their family's unique context. Previous research also underscores the value of personalised care, particularly where the weight stigma may strain the parent–professional relationship (Pozniak et al. 2024). One relevant approach to individualised care is family‐centred care (FCC), which fosters collaboration between families and HCPs through mutual trust, respect and an understanding of each family's unique context (Kokorelias et al. 2019; Kuhlthau et al. 2011). FCC, widely applied in paediatrics and nursing (Hriberšek et al. 2024), is recommended in paediatric weight management to support parental engagement and shared decision‐making (Farnesi et al. 2012). For parents, FCC may help them feel seen as individuals and reduce fears of being judged solely on their child's weight.

In Finland, public health nurses describe their approach to child weight management as family‐centred, yet they also report challenges such as occasionally ineffective counselling and limited multidisciplinary collaboration, often restricted to referrals (Mäenpää and Vuori 2019). The gap between parents' experiences in this study and public health nurses' perceptions of FCC warrants further investigation, particularly regarding how the challenges identified by nurses may influence parents' experiences in a less positive way. Evidence suggests that when care is genuinely family‐focused, it can improve outcomes for children, parents and overall family functioning (Janssen et al. 2025).

Second, our findings show that parents expect weight‐related encounters to protect the child's well‐being, body image and relationship with food. This protective instinct has been recognised in previous research (Åsberg et al. 2023; Eli et al. 2022; Gillison et al. 2014; Moyer et al. 2014) and is particularly understandable in the case of children with higher weight, as they are more likely to experience lower self‐esteem (Wang et al. 2009).

However, while the parents prioritised protecting their child from stigma or disordered eating, they also sought clear information and structured support to address health risks, low self‐esteem and social challenges like bullying. This reflects a tension between safeguarding emotional well‐being and managing physical health—an emotional and moral struggle also identified by Davis et al. (2018). Parents often described health care encounters as unresponsive, revealing a tension between weight management goals and supporting the child's emotional well‐being—a contradiction noted in prior research (Andreassen et al. 2013). HCPs should create space for parents to discuss this tension and receive pre‐service and in‐service training in strategies that help address emotions around a child's weight, enabling broader solutions beyond mere adaptation.

Our findings also show that parents want to be recognised as active, competent partners in their child's care—a key tenet of family‐centred care (Dempsey and Keen 2008). Nevertheless, many also feared judgement or blame for their child's weight, revealing experiences of weight stigma by association. This was reflected in how they described their involvement and their efforts to avoid being seen as inadequate caregivers. These accounts mirror broader cultural narratives that hold parents responsible for their child's weight, shaping ideals of ‘successful’ parenting (Quirke 2016) and prompting parents to prove their caregiving competence (Koivumäki and Jallinoja 2023).

In addition to societal attitudes, the health care system itself can contribute to stigmatising experiences (Ryan et al. 2024). Our findings resonate with previous research documenting parents' recurrent experiences of feeling held accountable by HCPs for their child's weight status (Eli et al. 2022; Gorlick et al. 2021; Hardy et al. 2019). Parents described how assumptions based solely on the child's weight led to feelings of blame and frustration. These experiences highlight the need to shift towards empowering parents and strengthening their self‐efficacy. A child's weight can deeply affect how parents perceive their own parenting (Lydecker and Grilo 2017), and research suggests that enhancing parental self‐efficacy can positively influence children's health behaviours, such as increased fruit and vegetable intake (Möhler et al. 2020). In addition to the content of counselling, the manner in which it is delivered is equally important. Parents should feel competent and supported, rather than inadequate or judged. Cultivating empathy has been identified as a key strategy for reducing the weight stigma in health care settings (Talumaa et al. 2022). There is a clear need for training programmes aimed at enhancing HCPs' skills in sensitive communication and promoting holistic approaches in paediatric weight management. In addition, raising awareness of weight stigma among HCPs should be integrated into both pre‐service education and ongoing training for those already working with families.

Thirdly, many parents in this study described health care responses as passive or unhelpful, an experience echoed in earlier research, which found that weight‐related guidance is ineffective (Saunders et al. 2023). Others, too, have reported HCPs being seen as overlooking weight‐related concerns, leaving parents uncertain about how to support their child's weight at home (Hardy et al. 2019). The experience of passivity is particularly noteworthy. While previous studies often portray parents as reluctant to engage in weight‐related care (Schroeder and Smaldone 2017; Thorstensson et al. 2018), our findings suggest a contrasting narrative: parents are actively seeking more supportive and responsive health care. This underscores a disconnect between HCPs' perceptions and parents' experiences regarding their role in care processes. Bridging this gap requires health care systems to offer timely and appropriate support within environments that are both safe and respectful—conditions that foster genuine engagement.

Parents' experiences in health care are largely focused on lifestyle, particularly nutrition. While many valued dietary counselling, others found it too generic or misaligned with their family's needs. Weight‐related counselling often lacks nuance, limiting its practical relevance (Eli et al. 2022; Ryan et al. 2024). Family‐based interventions, centred on nutrition, physical activity and behaviour, have a long history (Berry et al. 2004), with parents typically expected to take responsibility. Although parents often expect lifestyle counselling to address their child's weight, they struggle to integrate it into their daily life (Saunders et al. 2023). Given such interventions usually yield only modest, short‐term BMI reductions (Resnicow et al. 2024; Mead et al. 2017), health care must move beyond lifestyle‐focused approaches. A broader perspective is needed—one that considers parental well‐being, child‐rearing practices and family dynamics. Research also underscores the importance of addressing structural challenges like income and food insecurity, housing instability, limited access to education, legal issues and a lack of social support (Wahi et al. 2024). Echoing this, some parents in this study expressed a need for peer and everyday support.

Addressing higher weight in children within ‘real‐world’ settings remains a complex challenge (Rolke and White 2024), suggesting that proposed solutions should better account for this complexity. These findings underscore the importance of multiprofessional collaboration and a shift from weight‐focused care to holistic well‐being. To meet parents' needs, care for a child's weight should extend beyond health care to include psychological and social support through multidisciplinary collaboration. This is crucial for parenting and financial issues, which often fall outside health care's scope. In Finland, continuity with the same public health nurse fosters trust, and needs‐based family guidance can help allocate limited resources more effectively. The findings suggest that parents' expectations are shaped by the stigma surrounding higher weight in children. This stigma, which includes parents themselves being subject to judgment, has also been documented in previous research (Patel et al. 2023).

The findings highlight a dilemma parents face in weighing their child's best interests—whether to address the weight directly to reduce health risks or to protect the child from potential harm to body image and self‐esteem. This tension was evident in how parents perceived the support they received: while many acknowledged receiving help related to weight, it was often described as passive or poorly aligned with their family's specific needs.

### 5.1 Limitations

Interviewing on sensitive topics introduces selection bias towards parents comfortable discussing such issues, potentially overrepresenting openness about weight or strong views on health care services. Participants may also emphasise socially desirable aspects or avoid negative portrayals of parenting. Still, many parents spoke candidly about their own actions affecting the child's weight, indicating accounts were not solely aimed at demonstrating ‘good parenting’.

Fathers were underrepresented in this study, reflecting a broader trend across parenting and childhood weight‐related research, where they comprise only 17% of participants in observational studies (Davison et al. 2016). To increase fathers' involvement, innovative approaches are needed to make participation more accessible, relevant and engaging (Morgan et al. 2017). Equally important is including the voices of the children and adolescents themselves.

The data lacked ethnic diversity, as all participants were ethnically Finnish. This restricted exploration of culturally diverse perspectives, which may be shaped by factors like cultural values, language barriers and trust in health care. However, research suggests that parents across ethnic backgrounds often share similar concerns (Anderson et al. 2021; Gorlick et al. 2021).

As is often the case when recruiting participants, particularly on a sensitive topic, there is a risk of primarily reaching individuals who have already processed the issue to some extent. Moreover, recruitment through social media or newspaper may further introduce bias towards parents who are highly engaged or possess greater health literacy. However, a notable strength of this dataset is the diversity of educational backgrounds among participants, which suggests that the sample was not limited to highly engaged or health‐literate parents and therefore mitigates the risk of significant recruitment bias.

In addition, parents reported their experiences retrospectively, a few of them approximately 10 years after the health care encounters had taken place. While retrospective accounts may be influenced by recall bias or shaped by reconstructive memory, temporal distance can equally foster more considered and nuanced reflections.

Finally, the findings are context‐dependent, as child health monitoring in Finland relies on public health nurses through the established maternity and child health clinic system and school health services with regular family meetings.

## 6 Conclusion

This study highlights the complexity of providing sensitive and effective care for families navigating a child's higher weight. Parents' expectations are shaped by societal stigma and previous health care experiences, often resulting in a desire for individualised, emotionally safe and practically supportive care. A family‐centred approach that acknowledges stigma, strengthens parental self‐efficacy and reflects the multifaceted nature of weight is essential. Success should be measured not only by weight outcomes but by the quality of support provided to families in promoting the child's overall well‐being.

## Author Contributions

The first author (T.K.) was responsible for conceptualisation, research design (in collaboration with P.J.), data collection through interviews, obtaining ethical approvals, data analysis and drafting the manuscript. Co‐authors (M.K., S.A. and P.J.) contributed to the analysis and interpretation of the data, particularly in developing and refining the thematic framework. M.K. also supported the methodological design and provided supervision throughout the research process. All authors contributed to the critical revision of the manuscript and approved the final version.

## Funding

T.K. was supported by a grant for her PhD studies from the Alli Paasikivi Foundation. The funder had no role in content development.

## Ethics Statement

The Ethics Committee of the Tampere Region has given a supportive statement of the ethical acceptability of the proposed study (92/2021).

## Consent

Written or verbal informed consent was obtained from all participating parents prior to their involvement in the study. Participants were provided with detailed information about the purpose, procedures and confidentiality of the research, and they gave their voluntary consent in writing.

## Conflicts of Interest

The authors declare no conflicts of interest.

## Supporting information


**Data S1:** jan70525‐sup‐0001‐Supinfo01.pdf.

## Data Availability

The data generated and analysed during this study are not currently publicly available but will be archived in the Finnish Social Science Data Archive (FSD) following the completion of the project. Access will be granted in accordance with ethical and legal guidelines.
